# Visual perceptual learning is enhanced by training in the illusory far space

**DOI:** 10.1177/17470218241256870

**Published:** 2024-06-10

**Authors:** Antonio Zafarana, Carmen Lenatti, Laura Hunt, Munashe Makwiramiti, Alessandro Farnè, Luigi Tamè

**Affiliations:** 1 School of Psychology, University of Kent, Canterbury, UK; 2 University of Milano Statale, Milan, Italy; 3 Impact Team of the Lyon Neuroscience Research Centre, INSERM U1028, CNRS, UMR5292, University Claude Bernard Lyon I, Lyon, France

**Keywords:** Perceptual learning, visual learning, peripersonal space, extrapersonal space

## Abstract

Visual objects in the peripersonal space (PPS) are perceived faster than farther ones appearing in the extrapersonal space (EPS). This shows preferential processing for visual stimuli near our body. Such an advantage should favour visual perceptual learning occurring near, as compared with far from observers, but opposite evidence has been recently provided from online testing protocols, showing larger perceptual learning in the far space. Here, we ran two laboratory-based experiments investigating whether visual training in PPS and EPS has different effects. We used the horizontal Ponzo Illusion to create a lateralized depth perspective while participants completed a visual search task in which they reported whether or not a specific target object orientation (e.g., a triangle pointing upwards) was present among distractors. This task was completed before and after a training phase in either the (illusory) near or far space for 1 h. In Experiment 1, the near space was in the left hemispace, whereas in Experiment 2, it was in the right. Results showed that, in both experiments, participants were more accurate after training in the far space, whereas training in the near space led to either improvement in the far space (Experiment 1), or no change (Experiment 2). Moreover, we found a larger visual perceptual learning when stimuli were presented in the left compared with the right hemispace. Differently from visual processing, visual perceptual learning is more effective in the far space. We propose that depth is a key dimension that can be used to improve human visual learning.

## Introduction

Among other species, humans have a remarkable ability to learn from visual experience. Protracted training on specific elements of the visual scene improves our perceptual abilities through a process called perceptual learning (Gilbert et al., 2009; Sagi, 2011; Seitz & Dinse, 2007). Perceptual learning occurs in several if not all sensory domains: vision, touch, smell, hearing, and taste (Green et al., 2018). Research in vision has investigated visual perceptual learning using a variety of tasks (for a review see Dosher & Lu, 2017; Lu et al., 2011). Although the mechanisms driving visual learning remain debated (Ahissar & Hochstein, 2004; Bejjanki et al., 2011; Maniglia & Seitz, 2018), previous work has highlighted the high degree of specificity of such learning processes. Changes in performance are usually exclusive to the specific trained stimulus, or even the trained eye or visual field, without generalisation effects (Crist et al., 1997). Sigman and Gilbert (2000), for example, trained participants for several days in detecting a triangle with a specific orientation (target) embedded in an array of triangles with different orientations (distractors). They found that performance improved for the trained orientation and not the untrained ones and that the learning effects did not transfer to spatial locations close to the target one (Sigman & Gilbert, 2000). Thus, performance benefits are thought to be associated with a top-down cortical modulation from higher-level visual areas on the activity of early retinotopic areas (Sigman et al., 2005).

However, space is a very important dimension when processing stimuli in the external environment as their occurrence in different parts of the visual space (up, down, right, left) may determine different processes (Asanowicz et al., 2013; Berlucchi et al., 1974; Losier & Klein, 2004). Another important spatial factor is depth, that is, the distance at which stimuli occur with respect to the observer’s body, as well-established dissociations exist in terms of processing when stimuli are into the *peripersonal* or the *extrapersonal* space (Brozzoli et al., 2011; Rizzolatti et al., 1981b; Serino, 2019). The former represents the area that is close to our body while the latter represents the space that extends farther. Neurophysiological studies in monkeys revealed the existence of populations of neurons that specifically respond to both tactile and visual stimuli only when these are presented near the body (Duhamel et al., 1998; Graziano & Gross, 1993; Rizzolatti et al., 1981a, 1981b). Similarly, neuropsychological symptoms in humans, like visuo-tactile extinction, are known to increase at shorter distance from patients’ body surface (di Pellegrino et al., 1997; Làdavas, Di Pellegrino, et al., 1998; Làdavas, Zeloni, & Farnè, 1998). Several studies have thus investigated visual perception in the peripersonal space and converged in showing perceptual facilitation for stimuli close to the observers (Abrams et al., 2008; Ahsan et al., 2021; Blini et al., 2018, 2021; Dureux et al., 2021; Reed et al., 2006, 2010). Not only simple detection (Plewan & Rinkenauer, 2017) but also visual discrimination is faster when stimuli are presented near compared with far from the body (Blini et al., 2018). Participants are also more accurate in visual discrimination tasks according to (Ahsan et al., 2021) findings. Further research has shown that participants are more accurate as well as faster for high-level visual tasks, specifically face perception/discrimination when faces appear in the peripersonal compared with the extrapersonal space (Ahsan et al., 2021; Dureux et al., 2021). Moreover, attention resources during visual search tasks are biased toward the peripersonal space, as indicated by increased difficulty (higher reaction time) in disengaging attention from stimuli when these are near rather than far from the body (Abrams et al., 2008).

In the light of this large body of evidence favouring privileged visual processing in the peripersonal space, one would expect that visual perceptual learning should also benefit from this closeness advantage. Yet, the only available study, conducted online during COVID-19 outbreak, reported opposite evidence; namely, visual perceptual learning was actually more efficient in the extrapersonal space (Zafarana et al., 2023). In that study, participants who trained in a visual search task in the extrapersonal space became more accurate, whereas those who trained in the peripersonal space showed no significant changes in performance. However, in that study we used a vertical version of the Ponzo Illusion to create the depth perspective. This makes the upper part of the screen associated with the far space and the lower part with the near space, which according to Previc’s functional specialisation theory (Previc, 1990), are linked to higher visuomotor processing in the near/lower space and better visual search/recognition in the far/upper space. Moreover, as it was an online experiment, we could not monitor participants distance from the screen, display settings (e.g., luminosity and contrast), as well as their gaze. To overcome these potential limitations, here we ran a laboratory-based experiment focusing on the horizontal dimension of space in which stimuli can be presented, thus left and right sides.

**Figure 1.**
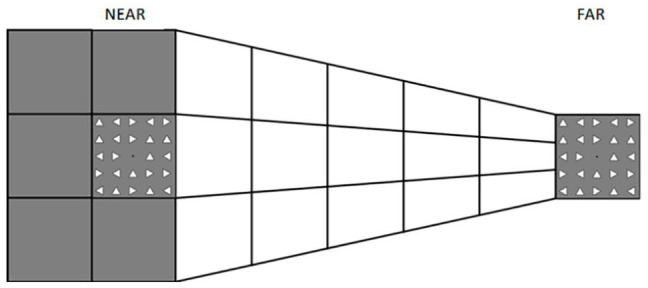
Depiction of the depth perspective in the image used to create the spaces for the stimuli presentation for the near (left) and far (right) conditions. In the near condition, the task was carried out on the square on the left side of the hemispace. Thus, stimuli (triangles) appeared on the left side while the matrix of triangles on the right side was always present without flashing. In the far condition, it was the other way around, stimuli were fixed on the left side of the screen and the visual search task was performed on the right.

**Figure 2.**
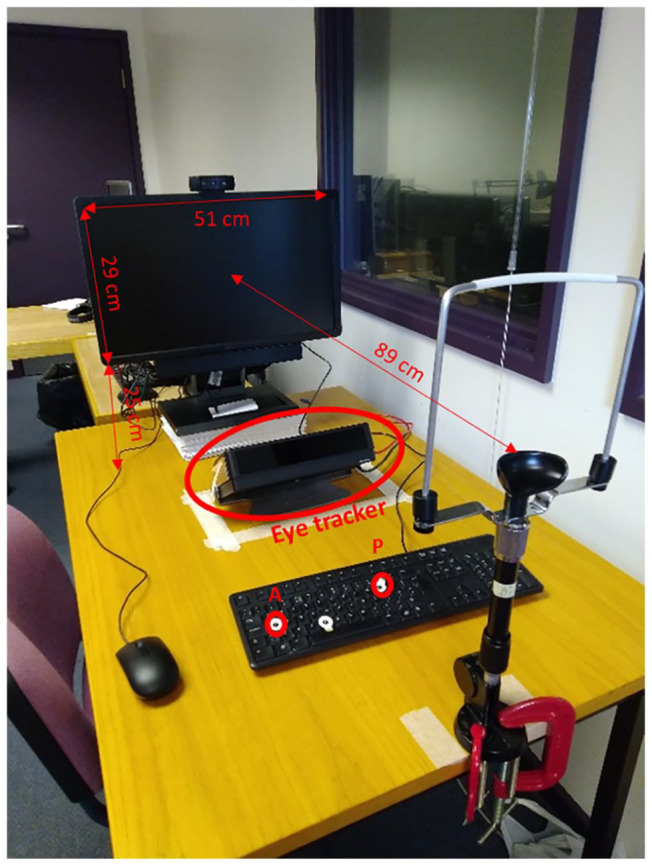
Experimental setup including computer, eye tracker, and chinrest.

In this study, we systematically examined whether visual perceptual learning has different effects in the peripersonal and extrapersonal space. We used a modified version of the horizontal Ponzo Illusion (see Figure 1) to create a depth perspective that would make one side as being either illusorily near or far from the observer (similar to Ahsan et al., 2021). Similar to our previous study (Zafarana et al., 2023), we tested participants on a visual search task in which they had to report whether a target object (i.e., triangle) in a specific orientation among 23 distractors was present or absent, both before and after a training phase. Critically, in both the testing and training phases, the visual display could illusorily appear either in the peripersonal or extrapersonal space. Two groups of participants were trained either in the near or far space for one of the four possible orientations (triangle pointing up, down, right, left). To control for any lateralized depth effect, the illusorily near and far conditions were associated with the left and right side of the display (Experiment 1) or the reverse (Experiment 2). In addition, we used an eye-tracker to monitor participants’ gaze during the training and testing phases (see Figure 2) to ensure participants were looking at the expected side of space.

If PPS visual advantage applies also to visual perceptual learning, we should expect an increment in performance for the trained orientation in the near and not (or larger than) in the far space. Alternatively, and in keeping with the recent results (Zafarana et al., 2023), if the visual advantage for visual perceptual learning applies to the EPS, we should expect an improvement in performance after training in the far, but not (or larger than in the) near space and that this learning effect would be specific (only for the trained orientation).

## Experiment 1

First, we investigated the effects of visual training on a visual search task in two groups of participants, one trained in the *peripersonal* space (near the body) and the other trained in the *extrapersonal* space (far from the body). The near space was presented in the participants’ left hemispace, whereas the far space was presented in the participants’ right hemispace. We measured participants’ performance in a visual search task before and after the training in both spaces. Note that differently from our previous study (Zafarana et al., 2023) the depth perspective here was created on the left and right hemispace rather than on top and bottom part of the hemispace. Also, at odds with our previous study, here the training lasted only 1 h and occurred in the same day rather than being distributed over 5 days.

## Materials and methods

### Participants

Forty-four participants (mean age = 23, range = 18–48; 35 females) took part in the experiment. Based on data from a previous study we conducted online (Zafarana et al., 2023), a priori power analysis was performed using G*Power version 3.1.9.7 (Faul et al., 2007) to estimate the sample size to compare the performance for the trained orientation before and after training. The effect size in that study was Cohen’s *d* = 0.88, which is considered large according to Cohen’s (1988) criteria. If the significance criterion is α = 0.05 and power = 0.99, the minimum sample size required for this effect size is *N* = 26 (total) for a two-tailed paired sample *t*-test. Hence, the resulting sample size of *N* = 20 per group is enough to test the research hypotheses. We slightly increased the number of participants to compensate for the reduced number of trials during this training (*t* = 1,200) compared with the previous study (*t* = 3,000). All participants had normal or corrected to normal vision. The study was approved by the ethics review committee at the School of Psychology, University of Kent and was carried out according to the principles of the 1964 Declaration of Helsinki as updated by (World Medical Association, 2013). Thirty-eight participants were right-handed (*M* = 95, range: 89–100) and four were left-handed (*M* = −91, range: −100 to −82). Handedness was determined using the Edinburgh Handedness Inventory with the laterality quotient (LQ) (Oldfield, 1971).

### Apparatus and stimuli

The visual search task was built in PsychoPy 3 (Peirce et al., 2019) and it was presented on a Dell U2312HM Monitor with a refreshing rate of 60 Hz and 1920 × 1080 pixels display resolution. Participants carried out the task in a laboratory room and were sitting at a distance of 89 cm from the screen, measured from their eyes. They also regulated the height of the seat to find a comfortable position to place their head on the chin rest (see Figure 2).

Visual stimuli consisted of a 5 × 5 matrix of 24 triangles with a fixation black dot positioned in the middle of the matrix. Each triangle had black outlines and a white fill. The sides of every triangle were 0° 27’ in length and there was a 0° 54’ distance from their centres; therefore, the matrix subtended 4.5° × 4.5°. Stimuli were presented on top of a white and grey background image (see Figure 1) which created a 2D depth perspective (Ponzo illusion) producing two illusory distances (near and far from the observer). The background image used, created a horizontal depth perspective resulting in near space on the left half of the screen and the far space on the right half. Participants’ task was to report whether the target was present or absent by pressing “P” or “A” respectively on a keyboard (standard QWERTY keyboard) using their hands.

### Eye-tracker

Participants’ gaze was monitored and recorded using the Tobii X120 eye tracker with a sampling rate of 60 Hz, continuously recording the gaze of both eyes through the experiment. Participants’ head was stabilised thanks to a chin/headrest in front of which the eye tracker was positioned, just below the screen (Figure 2). This was used to confirm that participants were gazing at the designated point of space, either near or far depending on the condition. Before commencing the experiment, the eye tracker was calibrated. Five circles, appeared randomly on the screen’s four corners and in the centre, were used as the calibration points.

### Design

**Figure 3.**
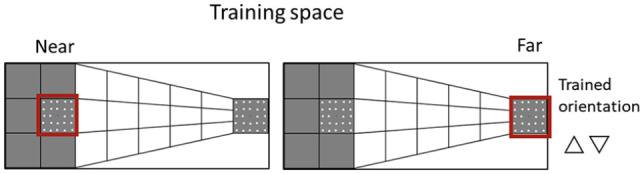
All possible experimental conditions. Participants were trained on one specific orientation (i.e., triangle pointing up or down) in the near (left) or far (right) space. The near and far spaces are highlighted by a red square in the depiction; however, this was not present in the experiment.

This was a mixed-subjects experimental design with three within-subjects factors and one between-subjects factor (see Figure 3). Participants trained in the near or far space and only on one specific orientation (up or down).

**Figure 4.**
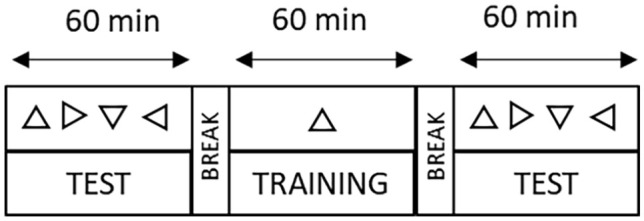
Timeline of the experiment including a testing phase lasting 60 min in which the four different triangle orientations were tested as a target in separate blocks. A training lasting 60 min in which only one orientation was used as a target. Finally, the last hour post-training testing phase in which all four orientations were tested as the target.

The experiment consisted of two testing phases and a training phase between them (see Figure 4). There were three within-participants factors: Orientation (Trained, Untrained), Time (Before, After), and Space (Near, Far). There was also a between-participants factor: Training (Near, Far). The trained orientation was counterbalanced across participants.

### Procedure

Participants gave written consent before starting the experiment in accordance with the procedure and protocols of the University of Kent. The experiment started with a testing phase and participants began the visual search task either in the near or far space according to the sequence order (i.e., A: near-far, B: far-near) and then they performed the same task in the opposite distance. In each test phase (before and after training), participants were presented with a target, a triangle pointing up, down, left, or right, at the start of each block (four in total, one for each orientation). Thus, in the phase before and after the training, participants completed four blocks in the near space and four blocks in the far space. Each block consisted of 150 trials, 20% of which were null, that is the target was not present, but it was present in the remaining 80% of the trials. There were a total of 1200 trials divided in 600 near and 600 far (1200 in each phase), whereas the training included 1200 trials in one space (near or far). Regardless of the phase, in all trials, there were two matrices of triangles, one in the near space and one in the far space. However, depending of the block, one matrix (e.g., the one in the near space) was changing from trial to trial, whereas the other one was fixed (e.g., the one in the far space) and participants were instructed to only look at one specific spatial location (near or far) according to the block. Each trial had a total duration of 3,000 ms and began with the presentation of a 300-ms visual stimulus (matrix of triangles flashing). Participants were then given 2,700 ms to answer before the next trial began. The stimuli of the following trial were presented even if the response was not given. The background image (Ponzo illusion) was statically displayed on the screen throughout the whole block (Figure 3). Participants indicated whether the target (e.g., triangle pointing up) was present (by pressing “P”) or absent (by pressing “A”) among the other triangles in the matrix. The target was presented 5 times at each of 24 possible locations of the 5 × 5 matrix. Each of the three phases lasted about 1 h as the total duration (roughly 3.5 h) depended on the participants since they were allowed to take a break every 75 trials (half block). Participants underwent a practice phase during which they learned how to perform the task. Once ready, they started the actual experiment. The visual task used was the same for all three phases (before-testing, training, and after-testing) except for the following aspects. In the training phase, participants were trained by repeating blocks in a specific target orientation (up or down) and only in one of the two spaces. This resulted in 2 possible conditions for each training group: orientation up and orientation down for the respective trained space (near, far). The order of the blocks near and far in both testing phases was counterbalanced. Finally, at the end of the experiment, participants received either credits or monetary compensation for their time, regardless of their performance.

### Analyses

We used the proportion of hits (target present and response “P”) and false alarms (target absent and response “P”) to calculate both the d-prime (d’) and the criterion (Swets et al., 1961). If no key was pressed, the response was classified as absent. When the false alarm rate or the hit rate was 0, their values were adjusted to 0.01; when the false alarm or hit rates were 1, their values were adjusted to 0.99. The d-prime was calculated using the formula: d’ = *z*(H) − *z*(FA) while for the criterion we used the following: c = −(z(H)+z (FA))/2. The d’ and criterion values obtained were entered as dependent variables in linear mixed-effects models. We decided to use linear mixed-effects models because they have been proven to be useful methods that take into account random effects such as inter-participant variability, allow generalisation across participants and factors and do not have the several variance-covariance limitations/assumptions of the repeated measures analysis of variance (rmANOVA). We followed a data-driven approach to create the model with the most complex random structure by adding each variable (orientation, time, space, training) step by step as a random effect and then testing the significance of each and keeping those that improved the model fit. The model fit, which indicates how well the model explains the data, was assessed by likelihood tests on the χ² values. We then evaluated the role of fixed effects (orientation, time, space, training) and their interactions. In the case of variables’ interaction effects, their impact was compared against a model including their main effects. These analyses were performed only on the data in the before and after-testing phases and not on the training phase. Raw data and analyses scripts are available on osf (https://osf.io/g23tb/).

### Eye tracker

We started by selecting the left gaze *x* and right gaze *x* coordinates as well as the left and right gaze *y* coordinates for all the trials when the stimuli were presented, and the eyes were open (could be detected). Then, we calculated the mean for both *x* and *y* coordinates and we used a Kernel Distribution Estimation Plot to present them thus, creating a colour map.

### Results

#### d-prime

Our null model, thus the model with the best random structure, had orientation and time as random effects. The model with the orientation and time interaction was significantly better than the one with their main effects, χ²(1, *N* = 44) = 5.31, *p* = .021, Cohen’s *d* = 0.29, 95% CI = [0.1, 0.47]. We found that participants’ performance after the training was significantly higher for the trained (*M* ± *SE* = 0.71 ± 0.08) compared with untrained (*M* ± *SE* = 0.55 ± 0.08) orientation, β = −.15, *SE* = 0.07, 95% CI = [−0.27, −0.02]. These results were confirmed by a post hoc test corrected for six multiple comparisons (Bonferroni–Holm corrected), *t*(43) = 2.97, *p* = .023, *d* = 0.29. The Time × Space interaction was tested against the null model including their main effects and the model fit was significantly better, χ²(1, *N* = 44) = 4.76, *p* = .03, Cohen’s *d* = 0.2, 95% CI = [0.1, 0.47], β = .14, *SE* = 0.06, 95% CI = [−0.01, 0.27]. However, post hoc tests corrected for multiple comparisons (Bonferroni-Holm corrected) did not show any significant differences between the levels of the variables (all *p* > .36). We also found that the Orientation × Time × Space interaction significantly improved the model fit, χ²(1, *N* = 44) = 14.05, *p* = .007. Nevertheless, the model’s summary showed only a significant Time × Space interaction, β = .25, *SE* = 0.09, 95% CI = [0.09, 0.45]. After training (both near and far), participants were significantly better for the trained (*M* ± *SE* = 0.78 ± 0.08) compared with the untrained (*M* ± *SE* = 0.55 ± 0.08) orientation, but only when the task was carried out in the far space, Cohen’s *d* = 0.42, 95% CI = [0.15, 0.69]. We also conducted a post hoc test corrected for 28 multiple comparisons (Bonferroni-Holm corrected) to further confirm the results, *t*(43) = 3.83, *p* = .02, *d* = 0.42. The performance on the near space after training (both near and far), was not significantly different between the trained (*M* ± *SE* = 0.64 ± 0.08) and untrained (*M* ± *SE* = 0.56 ± 0.08) orientations, *t*(43) = 1.20, *p* = 1.00, *d* = 0.15.

#### Criterion

Our null model, which included the best random structure, had time as random effect. The analyses showed a main effect of orientation, therefore the model fit with time as the fixed effect was significantly better than the null model, χ²(1, *N* = 44) = 4.43, *p* = .035, Cohen’s *d* = 0.12, 95% CI = [0.1, 0.22]. Participants were more conservative when carrying out the visual search task for the trained (*M* ± *SE* = 1.18 ± 0.08) compared with untrained (*M* ± *SE* = 1.11 ± 0.08) orientations, β = −.07, *SE* = 0.03, 95% CI = [−0.14, −0.004].

#### Eye tracker

Results from the eye tracker monitoring confirmed that participants were focusing on the quadrant they were instructed to look at through the experimental conditions. This assured that they were effectively performing the test while looking at the illusorily near or far space, accordingly to the instructions.

### Discussion

In Experiment 1, we found that participants performed better when tested in the far space in the trained orientations, regardless of whether they trained in the near or far space. This shows that the training in the far space led to improvements that were localised and specific to the trained space and orientation, whereas the training in the near space improved performance specific for the trained orientation, but this effect was only visible when tested in the far space. It is not clear if the difference between trained and untrained orientation after the training is driven by an increased sensitivity for the trained orientation, or a decreased sensitivity in the untrained orientation, or a compound of both. Indeed, the before and after comparison for the trained and untrained conditions did not differ, being only the after training conditions that differs. This effect is similar to what we found in a previous study (Zafarana et al., 2023), in which the training improved only in the far space. Here though, both the near and far trained groups showed improvement in the far space. One possible reason for this discrepancy may relate to the position where we created the illusory depth perspective (left and right) with respect to our previous work (top and bottom). Indeed, the left hemispace in which the illusory near space was created in this study may have played a special role in the learning process (Jewell & McCourt, 2000). Many studies have shown that healthy participants have a leftward bias, also called pseudo neglect (Bowers & Heilman, 1980), in several tasks (Charles et al., 2007; Nicholls et al., 1999). For instance, when participants have to determine the centre of a line they tend to bisect the line toward the left compared with the actual centre, which indicates that they perceive the left side of the line as longer (Jewell & McCourt, 2000). This bias has been attributed to the prevalence of visuospatial attention directed toward the left hemispace (Mattingley et al., 2004; Nicholls & Roberts, 2002). Based on these findings, it could be that the left hemispace assignment we made for the near and far space could have influenced participants’ performance. To control for this possibility and replicate the far space advantage in visual perceptual learning, we ran another experiment.

## Experiment 2

As mentioned, an aspect that could have led to Experiment 1’s results is the hemispace (left and right) in which the near and far conditions were respectively displayed. To test this possibility, in Experiment 2, we reversed the near and far conditions with respect to the hemispace in which the stimuli were presented (i.e., near to the right and far to the left hemispace). Thus, a group of participants trained in the right hemispace (near) and the other group in the left hemispace (far).

### Participants

Forty participants (mean age = 24, range = 19–49; 29 females) took part in the experiment. Based on the data from Experiments 1 (*N* = 42), we carried out a priori analysis using G*Power version 3.1.9.7 (Faul et al., 2007) to estimate the necessary sample size for the comparison between trained and untrained orientation after the training in the far space. The effect size in the two experiments was 0.42, which corresponds to medium, based on Cohen’s (1988) criteria. When the significance criterion is α = .05 and power = 0.80, the estimated sample size is *N* = 47 (thus 23/4 participants per group) for a two-tailed paired sample *t*-test. However, due to the high difficulty of the recruitment processes during the covid pandemic we were able to reach 20 participants in each group, slightly below the expected number. All participants had normal or corrected to normal vision. The study was approved by the ethics review committee at the School of Psychology, University of Kent and was carried out according to the principles of the 1964 Declaration of Helsinki as updated by World Medical Association (2013) except for registration in a database.

### Methods and materials

#### Visual search task

**Figure 5.**
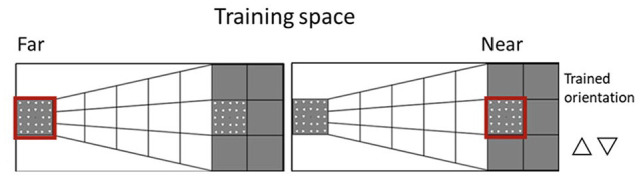
Portrayal of the stimuli presentation for the far (left) and condition near (right) spatial conditions. The participants received training on a single orientation (a triangle pointing up or down) in either the near or far space. In the illustration, a red square designates the near (right) and far (left) spaces; in the experiment, this square was absent.

The stimuli used in this Experiment were identical to the ones used in Experiment 1, except for the location of illusory depth. Instead of having the near space on the left half of the screen and the far space on the right half, it was the other way around. Therefore, the new background image created a depth perspective that resulted in the near distance on the right hemispace and far distance on the left hemispace (Figure 5).

#### Procedures and analyses

The procedures for this experiment were identical to the one used in Experiment 1 with the following exceptions. Participants carried out the training in the near space on the right half of the screen and training in the far space on the left half of the screen. The analyses carried out were identical to Experiment 1.

### Results

#### d-prime

Our null model, the model that has the best random structure included time as random effect. The model with the orientation and training interaction had a significantly better fit than the one consisting of their main effects, χ²(1, *N* = 40) = 4.55, *p* = .03, β = −.16, *SE* = 0.07, 95% CI = [−0.29, −0.01]. However, post hoc Bonferroni-Holm corrected multiple comparisons showed that there was no significant difference between the levels of the two variables. We also found that the model fit is significantly higher when the Orientation × Time × Training interaction is included compared with the model with their main effects, χ²(1, *N* = 40) = 10.65, *p* = .03. Despite this, the model summary showed no significant effect or interaction.

Finally, the Orientation × Time × Space × Training interaction was tested against the model including only their main effects and the former model fit was significantly better, χ²(1, *N* = 40) = 25.13, *p* = .009. However, the model summary indicated only a significant Time × Space × Training interaction, β = .62, *SE* = 0.20, 95% CI = [0.25, 0.98]. Participants who trained in the space far had a significantly better performance for the trained (*M* ± *SE* = 0.87 ± 0.11) compared with the untrained (*M* ± *SE* = 0.55 ± 0.12) orientation after the training and when the task was done in the far space. We conducted 2 post hoc paired t tests based on our hypotheses (Chatham, 1999; Kwon, 1996; Ruxton & Beauchamp, 2008), corrected for multiple comparisons (Bonferroni-Holm corrected) which confirmed the d-prime was higher for trained rather than the untrained orientation for the group that trained in the far space *t*(19) = 3.03, *p* = .014, Cohen’s *d* = 0.63, 95% CI = [0.17, 1.10]. However, participants who trained in the near space did not show a significant difference between the trained (*M* ± *SE* = 0.50 ± 0.11) and untrained (*M* ± *SE* = 0.61 ± 0.11) orientations in the far space after the training, *t*(19) = −1.08, *p* = .59, *d* = −0.22, 95% CI = [−0.51, 0.6]. Finally, an exploratory analysis based on the observed results revealed that there was also a significant improvement of participants’ performance when they did the training in the far space for the trained orientation from before (*M* ± *SE* = 0.52 ± 0.10) to after (*M* ± *SE* = 0.87 ± 0.11) the training, but only when the task was carried out in the far space, *t*(19) = 2.54, *p* = .02, Cohen’s *d* = 0.57.

#### Criterion

The analyses revealed that the model with the best random structure had only time as random effects, thus we used it as our null model. We tested the models including the fixed effects for orientation, time, space and training and their interactions but we found no significant model (*p* > .05).

#### Eye-tracking

Similar to Experiment 1, participants were focusing on the quadrant they were instructed to look at through the experimental conditions. This assured that they were effectively performing the test in the near or far spaces accordingly to the instructions.

### Discussion

Experiments 2 showed, as in Experiment 1, that the participants who trained in the far space had a significant improvement in their performance (higher d-prime) for the trained orientation, but only when tested in the far space. However, the group who trained in the near space did not show any significant change after training. Therefore, while the results indicate a robust effect of visual perceptual learning in the far space, they emphasise that the training is distance specific. These findings may imply that the hemispace in which the training is performed plays a role on the effectiveness and generalisability of the training.

## General discussion

In this study, we investigated if visual perceptual training has different effects based on whether the stimuli are presented near or far from the body (*peripersonal* and *extrapersonal* space). In Experiment 1, participants had the far space in the right visual hemispace, and in Experiment 2, they had it in the left visual hemispace. In both experiments, one group of participants were trained on a visual search task where they looked for a triangle in a specific orientation in the near space, whereas the other group of participants trained in the far space. When the near space was on the left side of the screen, we found that both the participants who trained in near and far spaces after the training had a significantly better performance (for the trained orientation) when tested in the far space (Experiment 1). When the near space was on the right side of the screen and the visual search task was carried out in the far space only the group of participants who trained in the far space showed a better performance (for the trained orientation) after the training in the far space (Experiment 2).

These findings bring support to those reported in a recent study conducted online using a similar paradigm (Zafarana et al., 2023). In addition to replicating initial evidence of far gain, this study provides robust additional evidence of such an advantage by offering further internal replication of such far learning advantage. Although we find evidence that the hemispace where training is performed might affect the magnitude of the far space advantage, it is the depth dimension that really leads the visual perceptual learning effect.

**Figure 6.**
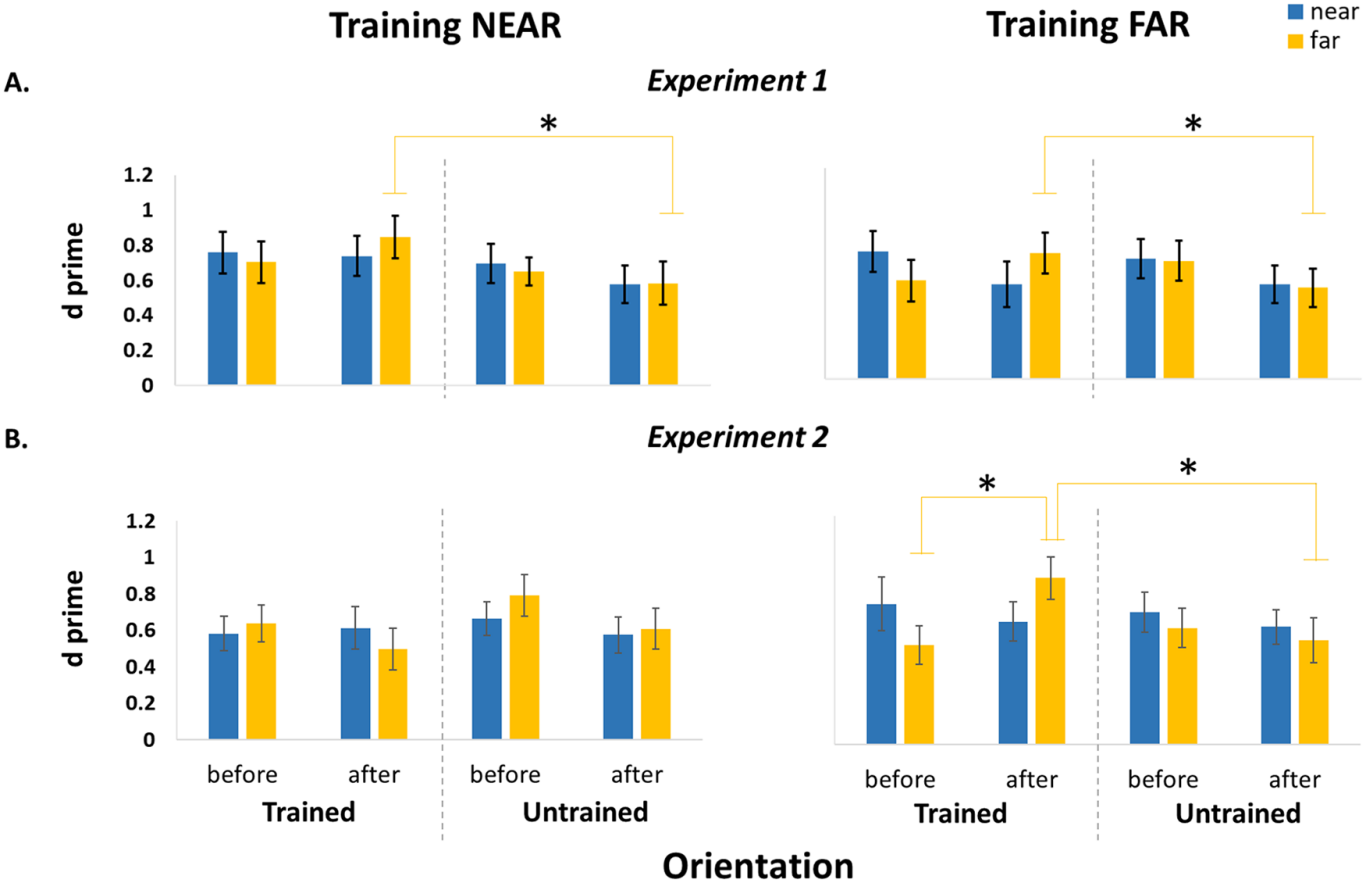
Illustration of the results for the Experiment 1 (A) and 2 (B). Bar charts showing the d-prime values for the participants who trained in the near space (left panel) and those who trained in the far space (right panel). The data presented in the chart are divided into Orientation (trained and untrained) and Space (near in blue and far in yellow). Error bars represent the standard error of the mean (± SEM). *p < .05.

Overall, the results of the present study show that visual training always produces an improvement in the far space: when participants performed the visual training in the far space and sometimes also when they did the training in the near space. In addition, the key factor that seems to drive the greater effectiveness of the training in Experiment 1 (whereby both the near and far training groups improved in the far space) seems to derive from the hemispace in which the training is performed. This notion is also supported by Experiment 2 results which showed that training in the near space on the right hemispace led to no significant changes. Indeed, the left hemispace seems to be more effective in promoting such visual learning processes (see Figure 6). These results are in accordance with the literature suggesting an attentional bias toward the left hemispace (Jewell & McCourt, 2000; Mattingley et al., 2004; Toba et al., 2011). In Experiment 1, it was observed that even if there was an attentional bias, it did not result in an improved performance in the near space. Based on the results from a previous study (Abrams et al., 2008), showing a higher difficulty to disengage attention from stimuli in the space near us, it might be assumed that the attentional advantage toward the left hemispace was not enough to produce a significant improvement in performance in the peripersonal space. However, in Experiment 1, we found that training in near space induced a learning effect in the far space. Despite the absence of any observable initial disparities in the performance between the two spatial areas (i.e., near and far), it is conceivable that attentional processes played a more prominent role during perceptual training in the extrapersonal space. This could be attributed to the fact that participants were able to more readily disengage and reorient their attention in that space.

It must be noted that the use of the Ponzo Illusion might have led the participants to perceive the triangles in the far space as bigger compared with those in the near space, due to size-constancy mechanisms (Sperandio & Chouinard, 2015), even though, both the physical and the retinal sizes were equal between the triangles in the near and far spaces. This could have played a role, favourably improving the learning phase. Note however that if this were the case, it would have been a peculiar effect that only affects the learning phase, since before undergoing the training, there was no significant difference in the participants’ performance between the illusory near and far (i.e., illusory greater size) spaces.

In summary, we found that training in the far space leads to significant improvements in performance on a visual search task in the trained location that is specific to the trained orientation. However, training in the near space results in a better performance but only in the far space or no change at all, based on whether it is carried out on the left or right hemispace, respectively. Therefore, our results suggest that visual training is more effective when performed in the illusory far space and the left hemispace. Such advantage can derive from a combination of different mechanisms. Stimuli in the PPS are processed faster and more accurately compared with those in the EPS (Ahsan et al., 2021; Blini et al., 2018; Plewan & Rinkenauer, 2017; Reed et al., 2006). However, this PPS advantage can lead to apparently contradictory results in which it is more difficult to disengage attention from stimuli that are close rather than far from us (as shown in Abrams et al., 2008). In the current study, attentional processes might have been deployed differently in the two distances during the training phase, thus leading to a better learning in the far compare to the near space. The results obtained in this study converge with those found in a previous work (Zafarana et al., 2023) while overcoming some limitations, therefore confirming that, in visual tasks, closer may not always be better.
